# Quantitative Analysis of Polymers by MALDI‐TOF Mass Spectrometry: Correlation Between Signal Intensity and Arm Number.

**DOI:** 10.1002/jms.70023

**Published:** 2026-01-01

**Authors:** Mete‐Sungur Dalgic, Sourabh Kumar, Steffen M. Weidner

**Affiliations:** ^1^ Bundesanstalt für Materialforschung und ‐prüfung ‐BAM Berlin Germany

**Keywords:** MALDI TOF MS, polymers, quantification, topology

## Abstract

The signal intensities of linear and star‐shaped poly(L‐lactides) (PLA) and poly (ethylene oxides) (PEO) were compared to determine the influence of the number of arms on the ionization in matrix‐assisted laser desorption/ionization time‐of‐flight (MALDI‐TOF) mass spectrometry. In this study, a variety of blends were prepared and investigated, including binary and ternary combinations of linear and star‐shaped polymers with similar molecular masses. The focus was on examining their intensity ratios. In equimolar binary PLA blends, polymer stars were observed to exhibit higher intensities than their linear counterparts. This result was supported by experiments with equimolar ternary PLA blends, which clearly demonstrated an intensity dependence on the number of polymer arms. It was observed that four‐arm PLA exhibited higher intensities than three‐arm PLA. A similar trend was observed in investigations involving acetylated polymer end groups, suggesting that differences in ionization are primarily influenced by polymer architecture rather than end groups. In order to validate this assumption, the binding energies for [polymer‐K]^+^ adduct ions utilizing the most stable geometry obtained from GOAT (Global Optimizer Algorithm) were calculated, revealing that star‐shaped lower mass oligomers have slightly higher binding energies.

## 1 Introduction

MALDI‐TOF mass spectrometry is a valuable tool for characterizing synthetic polymers with regard to their molecular masses, structures, and end groups [1, 2, 3]. However, quantitative analysis with this technique remains a difficult task due to the signal dependence on various instrumental and chemical parameters [4, 5, 6]. For instance, instrumental parameters such as detector voltage and delay time can significantly impact mass signal intensities [7, 8]. Chemical parameters, such as polymer dispersity [9], the choice of matrix and cations, and their ratio [10], influence the quality of the obtained MALDI MS spectra. Another parameter that drastically affects the results of MALDI‐TOF MS is the applied sample preparation method [7, 11, 12]. In the case of solvent‐based methods, the careful selection of solvents is crucial, as incomplete dissolution or segregation of components can negatively impact the resulting data [7, 10, 13, 14, 15]. Solvent‐free sample preparation techniques can be used as an alternative to avoid such effects [12, 16, 17], but can cause additional problems regarding homogeneity and stability, especially with polymer blends [18]. Other issues, such as signal discrimination due to preferential ionization of one polymer in a copolymer or polymer mixture because of structural differences and/or different molecular masses, can cause a loss of information and may lead to the complete disappearance of analyte signals [9, 19].

The influence of the end groups on the ionization in MALDI‐TOF mass spectrometry has been reported in several studies, with contradictory results [20, 21, 22, 23]. These studies show that cation attachment depends on the structure of both the end groups and the polymer. With a few exceptions, such as when ionic end groups (e.g., ammonium) are present, polar polymers revealing different heteroatoms will be ionized by statistical (random) ion adduct formation at any monomer unit of the polymer chain. Nonpolar polymers with more polar end groups offer a specific site for attachment at their end groups.

In contrast, the effect of polymer architecture on MALDI‐TOF MS results remained largely unexplored. Recently, we demonstrated that cyclic PLA species exhibit slightly higher intensities than linear PLA counterparts in blends [24]. At this time, it was not possible to provide a reasonable explanation for this result. Neither the desorption nor the ionization processes in MALDI could be ruled out. To eliminate the effects of desorption, electrospray ionization (ESI) can be used instead. However, this technique causes other problems, mainly due to solvent desorption and overcharging of analytes. Thus, it might provide some insight into conformational effects of polymers with different topologies in the gas phase but cannot serve as an alternative, as demonstrated by the following examples.

Trimpin and Clemmer were the first who reported strategies that provided the structural signatures to recognize minor differences in blends and copolymers [25]. In another paper, De Pauw et al. applied IMS to experimentally derive structural information about cationized linear and star‐shaped poly‐ε‐caprolactones as a function of their charge state and chain length [26]. Through theoretical modeling, they identified two major conformations: (1) near‐spherical conformations whose sizes are invariant with the polymer topology for long and lightly charged chains and (2) elongated conformations whose sizes vary with the polymer topology for short and highly charged chains. However, most of these studies were limited to polymers with lower molecular masses (< 2000 Da). In 2015, Grayson et al. investigated three poly (ethylene glycol) (PEG) architectures with closely related average molecular weights of about 9000 Da: a linear PEG, an unevenly branched miktoarm star PEG, and an evenly branched homoarm star PEG [27]. In their study, the +7 charge state with Cs^+^ cations generated by ESI was used. They found that due to electrostatic repulsion forces, different charge states can result in very different gas‐phase conformations. Therefore, structural comparisons must utilize the same MW range, cation, and charge state.

Unlike ESI, MALDI TOF MS almost exclusively generates singly charged ions. Therefore, conformational changes based on different charge states should not occur. Unfortunately, there are only a few reports on the application of MALDI‐IMS available in the literature [28, 29]. In 1995, von Helden et al. were the first to couple a MALDI source to an IMS (called “ion chromatography” at the time) [29]. Using molecular mechanics methods, they discovered that the oxygen atoms on the PEG units (from *n* = 9 to 17) surrounded the Na^+^ ion, forming a crown ether‐type ring of five oxygens with several others above and below this ring. This finding is remarkable because these effects could have occurred either in the gas phase after desorption in the MALDI source or during sample preparation in the solid phase as the solvent evaporated. The latter possibility is supported by the “lucky survivor model” established by Karas et al. [30]. It postulates that analytes are incorporated in the matrix crystals with their respective charge states preserved from solution [31].

In this paper, we extended our previous MALDI‐TOF MS experiments of (linear) polymer blends on various binary and ternary systems consisting of linear and star‐like polymers. Polymer stars are particularly interesting due to their unique structure, which consists of a central core to which a different number of arms are attached. This results in distinct physical and chemical properties compared with those of their linear counterparts. Due to their lower melting and glass transition temperatures, crystallinity, and more compact structure, polymer stars are suited for example for medical applications such as drug and gene delivery, as well as effective drug encapsulation and release [32, 33, 34, 35].

Several studies have investigated polymer stars (dendrimers) using MALDI‐TOF MS; however, no comparisons have been made with linear polymers [36, 37, 38]. In an early paper, Xu et al. reported on the ability of poly (ethylene oxide) (PEO) chains to solvate metal ions [39]. On the basis of these results, Gnanou et al. commented that star‐shaped PEO can be expected to act as a solubilizing agent for inorganic salts in organic media [40].

**SCHEME 1:**
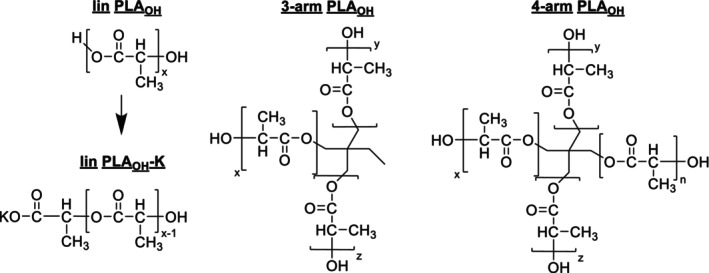
Structures of the linear, the three‐arm and the four‐arm PLA_OH_.

**SCHEME 2:**
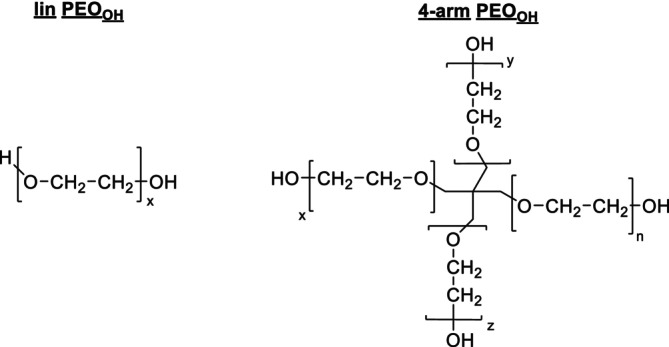
Structures of the linear and the four‐arm PEO_OH_.

No systematic study has yet been conducted on how topological variation influences ionization and desorption behavior in the MALDI process. To clearly attribute differences in MALDI spectra to topological variation, we examined linear and starlike polymers from two classes of polymers with identical monomer and end group structures and molar masses. We extended the investigation to include identical polymer blends after acetylating the PEO‐ and PLA‐hydroxyl end groups to determine whether the observed results were caused by the polymer architecture or the end groups. Their structures are shown in Schemes 1 and 2.

Finally, Kohn–Sham density functional theory (DFT) calculations were performed to calculate binding energies of the [polymer/K]^+^ adduct ions formed by linear and star‐shaped PEOs. These binding energies may provide a reliable estimate of the stability of such ions.

## 2 Experimental Section

### 2.1 Materials

L(‐)‐Lactide (LA) and *trans*‐2‐[3‐(4‐*tert*‐butylphenyl)‐2‐methyl‐2‐propenylidene] malononitrile (DCTB) were supplied by Tokyo Chemical Industry Co. Ltd. (Tokyo, Japan). Tin (II) 2‐ethylhexanoate (SnOct_2_), pyridine, acetic anhydride, potassium trifluoroacetate (KTFA), and 1,1,1‐tris (hydroxymethyl)propane (TMP) were obtained from Sigma‐Aldrich (Taufkirchen, Germany). Four‐arm PEO_OH_ was purchased from Creative PEGWorks (Chapel Hill, United States). Toluene, n‐hexane (HPLC grade), and pentaerythritol (PENT) were purchased from Carl Roth (Karlsruhe, Germany). Linear PEO_OH_ was supplied by PSS (Polymer Standards Service GmbH—a part of Agilent, Mainz). 1,4‐Dioxane was obtained from Th. Geyer (Renningen, Germany).

The molecular masses of all the polymers are listed in Table S1 (Supporting Information).

The acetylation of the PLA stars has been described elsewhere [23]. The acetylation of the linear PLA and the star‐shaped and linear PEO_OH_ polymers was conducted in a similar way.

### 2.2 Measurements

MALDI‐TOF MS: For sample preparation, 50 μL of DCTB (TCI, Japan) solution (20 mg mL^−1^ in chloroform) was premixed with 5 μL of a KTFA (Sigma‐Aldrich) solution (5 mg mL^−1^ in THF) and 20 μL of polymer solutions (1 mmol L^−1^ in chloroform). For binary blends, mixing ratios of 50/50, 80/20, and 20/80 were analyzed. For ternary blends, only equimolar ratios were prepared. All sample spots were prepared using an airbrush sprayer (Infinity CR Plus, Harder & Steinbeck, Germany). Three spots were measured for better reproducibility. After baseline subtraction and smoothing (Sawitzki‐Golay), mass spectra were evaluated.

MALDI‐TOF MS experiments were carried out in an Autoflex maX mass spectrometer (Bruker Daltonik GmbH, Bremen, Germany) with a 355‐nm Nd:YAG laser operating at 2000 Hz. Each mass spectrum was recorded in linear mode, and 8000 laser shots from different randomly chosen positions on the sample spot were collected.

## 3 Computational Details

Kohn–Sham DFT calculations with the range‐separated hybrid meta‐GGA functional **B3LYP D4** as implemented in ORCA 6 were performed [41, 42]. **B3LYP** is a hybrid GGA, and D4 is Grimme's atom‐pairwise dispersion correction integrated into the functional [43]. A triple‐ζ valence basis with one set of polarization functions (**def2‐TZVP**) together with the matching universal Coulomb‐fitting auxiliary basis **def2/J** is used to enable the RI‐J approximation for the Coulomb term [44]. The def2/J family is the recommended “*AuxJ*” fitting basis for def2 orbital bases in ORCA. Exact exchange was accelerated with the **RIJCOSX** algorithm, which yields substantial speedups for hybrid DFT [45]. DEFGRID3 was preset for all calculations, which sets tighter, XC and COSX grids (AngularGrid/COSX schemes and IntAcc values as defined by DEFGRID3) for robust energies and gradients [46]. Self‐consistent‐field convergence used the TightSCF protocol, which tightens the energy, density, DIIS error, and orbital‐gradient thresholds to reduce numerical noise in during geometry optimizations. An initial guess of the geometry is provided by ORCA's GOAT (Global Optimizer Algorithm) [47, 48] to explore conformational minima with the GFN2‐xTB semi‐empirical model [49]. GOAT performs numerous randomized local optimizations and generates a ranked ensemble of unique minima, ideal for inexpensive and pre‐screening of possible stable conformations before high‐level DFT calculations. The binding energy is calculated for [polymer‐K]^+^ adduct ion using the most stable geometry obtained from GOAT calculations.

## 4 Results and Discussion

### 4.1 Neat PLA Samples

**FIGURE 1:**
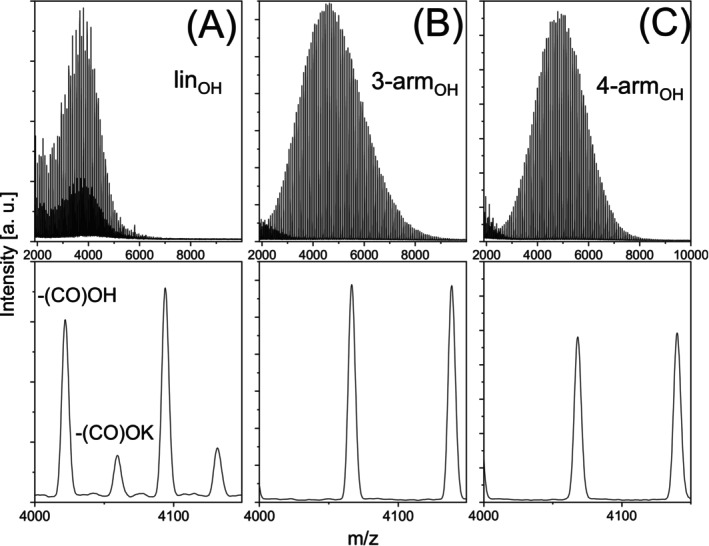
MALDI TOF mass spectra of linear (A), three‐arm (B) and four‐arm PLA_OH_ (C), and insets showing the region from *m/z* 4000 to 4150; −(CO)OK indicates the peak of the lin‐PLA_OH_‐K (see also Scheme 1).

The first set of experiments focused on the analysis of PLA polymers. In Figure 1, the mass spectra of PLA polymers with hydroxide groups are presented. The corresponding mass spectra of the acetylated polylactides are shown in Figure S1 in the supporting information (SI). The most interesting result from Figure 1 is the occurrence of two peak series in the spectra of the linear product. In addition to the expected linear structure with HO/COOH end groups, another series appeared with a distance of +38 Da. Since ionization was achieved by adding a potassium salt, this series can be attributed to an exchange of COOH for COOK end groups, which was recently observed when investigating other PLA polymers with carboxyl end groups [50].

These potassium “salt” end groups were not found in any of the starlike PLAs, because they do not have COOH end groups. A similar behavior was found for the acetylated products (Figure S1). There, the intensity of the potassium salt end groups is significantly higher. Moreover, a second, weak series of peaks in the spectrum of the four‐arm PLA_ac_ became visible (indicated by an #) representing incomplete acetylation of its end groups. As shown in a recent paper, its concentration could be calculated by means of chromatography and amounts to less than 3% [23].

### 4.2 Binary Blends of Linear and Starlike PLA

**FIGURE 2:**
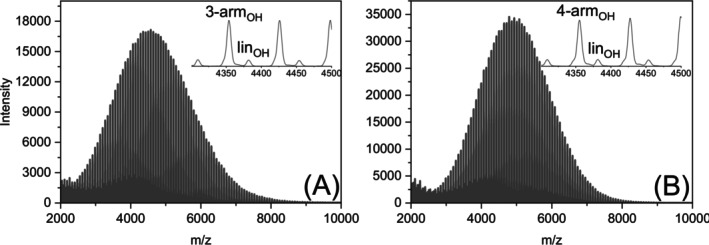
MALDI TOF mass spectra of equimolar binary blends of linear PLA_OH_ with three‐arm PLA_OH_ (A) and four‐arm PLA_OH_ (B).

The neat samples were used to prepare binary PLA blends with different mixing ratios followed by MALDI‐TOF MS analysis. Figure 2 shows the mass spectra of the equimolar blends of linear PLA_OH_ with three‐arm PLA_OH_ (A) and linear PLA_OH_ with four‐arm PLA_OH_ (B). Similar MALDI TOF mass spectra were recorded from identical blends of the acetylated species (lin‐PLA_ac_ with three‐arm PLA_ac_, and lin‐PLA_ac_ with four‐arm PLA_ac_), which are also shown in the SI (Figure S2).

As shown in Figure 2, in equimolar binary blends, the intensities of each PLA_OH_ star were about seven times higher than those of the linear PLA_OH_. Similar intensity differences were found in the spectra of the acetylated blends. There, the intensities of the starlike PLA_ac_ were about three (three‐arm PLA_ac_/lin PLA_ac_) and five times (four‐arm PLA_ac_/lin PLA_ac_) higher than those of linear PLA_ac_ (shown in Figure S2). At first glance, the intensity ratios seemed to be significantly higher than that of the OH‐terminated PLAs. However, it is important to note that over 50% of the lin‐PLA_ac_ chains contain COOK end groups, which is much higher than for lin‐PLA_OH_. Whereas a good quantification of both linear structures in the blends with the three‐arm PLA_ac_ was achieved (Figure S2A), the additional COOK series of the linear PLA_ac_ in the blend with the four‐arm star became only visible as a small shoulder on the right side of the four‐arm PLA_ac_ peak (indicated by an asterisk in Figure S2B). The two structures could not be clearly separated, so only a rough estimation could be made. Taking into account the summarized intensities of both linear species, our results again show that the structure of the end groups does not significantly affect ionization, but topology does.

There are several factors that may explain the significantly higher intensities of the star‐like species. Oxygen atoms are expected to serve as binding sites for cation coordination as the adduct formation in the MALDI ionization process is similar to the complexation of (alkali) cations in solution [28, 51, 52, 53]. As previously mentioned, polymer stars may have a higher capacity to complex ions than linear polymers [38, 39]. This is likely because star‐like polymers have a higher density of oxygen atom binding sites enabling the polymers to bind cations more easily in a cooperative manner as they approach each other [38, 39]. Therefore, the higher density of coordination sites in stars is presumed to be responsible for more effective complexation. This effect might be amplified due to higher flexibility and reduced entanglement of the star arms as compared with linear polymers, which can facilitate ionization [54, 55]. The same explanation applies to the slightly higher intensity of cyclic chains compared with linear chains, which has been reported recently [24].

Another explanation may be that PLA stars co‐crystallize differently with the matrices because they have a lower hydrodynamic volume than their linear counterparts [56]. From the literature and our own experiments using size exclusion chromatography (SEC) experiments combined with triple detection (light scattering, viscometry, and refractive index), it was known that the hydrodynamic volume of cyclic PLA is of two‐thirds that of the corresponding linear chains [57]. However, as described in the “Experimental Section”, all sample spots were prepared using the airbrush technique, which involves nearly complete evaporation of the solvent before deposition. Therefore, the coordinative insertion of a potassium cation into the polymer molecule must have occurred before or during air spraying and requires the partial dissociation of the potassium salt (KTFA) in the organic solvent (THF). Although such effects have been known for a long time [58, 59], our assumption is highly speculative since the stability of these solvent‐formed complexes after spot spraying and by laser ionization/desorption in the MALDI source cannot be estimated at this point.

Finally, as previously mentioned and shown in Figures 2 and S2 the formation of additional ion adducts with the –COOK end groups (PLA_OH_‐K) may also slightly contribute to the lower intensity of the linear polymers and thus to the overestimation of the intensity of the stars.

### 4.3 Binary Blends of PLA Stars

**FIGURE 3:**
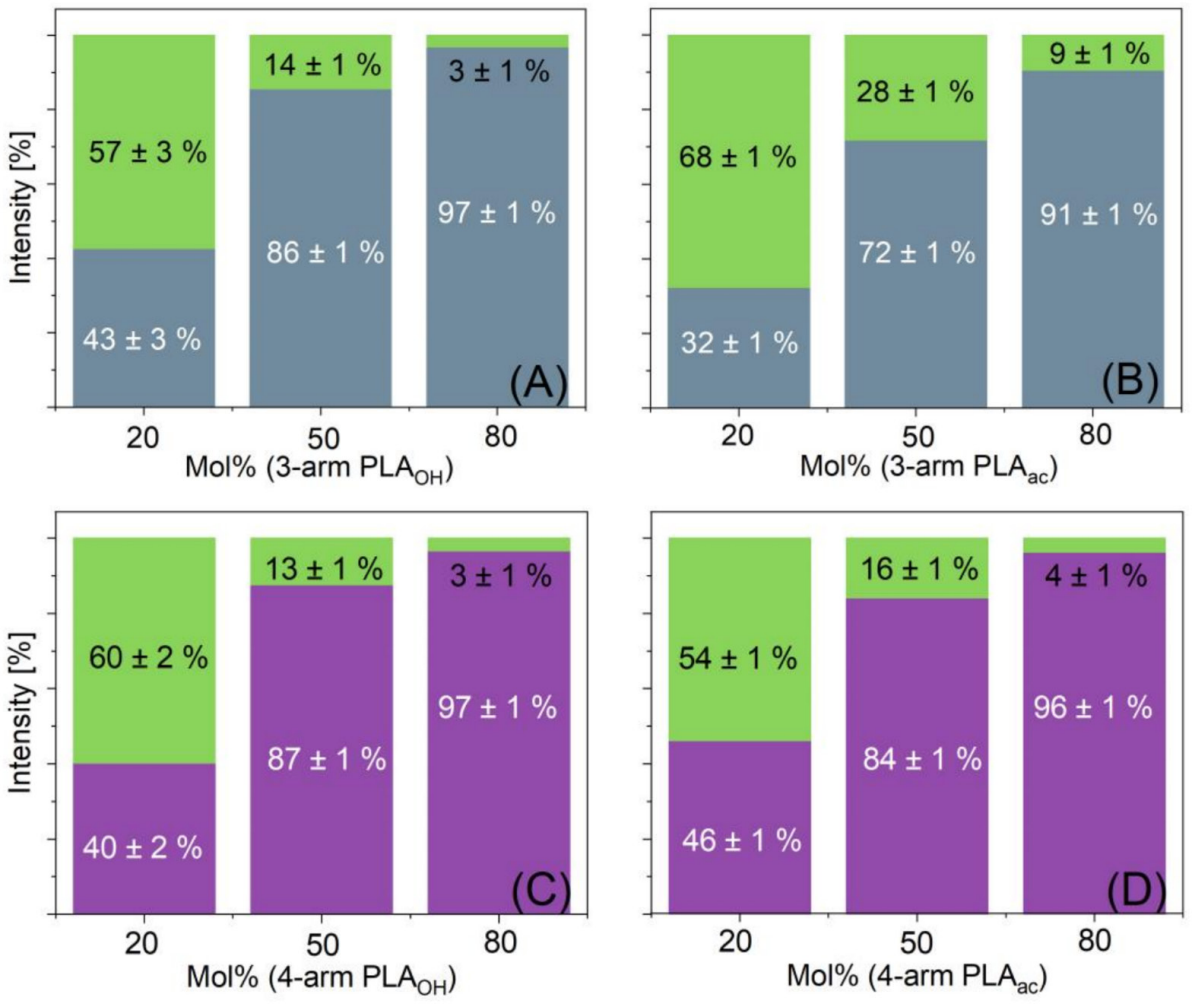
Intensity ratios of three differently composed binary blends: (A) linear PLA_OH_ (green) with three‐arm PLA_OH_ (gray), (B) its acetylated analogues, (C) linear PLA_OH_ (green) with four‐arm PLA_OH_ (violet), and (D) its acetylated analogues.

The strong influence of the number of arms was also proven by directly comparing three‐ and four‐arm PLA stars in binary blends with different compositions (20/80, 50/50, and 80/20) (Figure 3).

**FIGURE 4:**
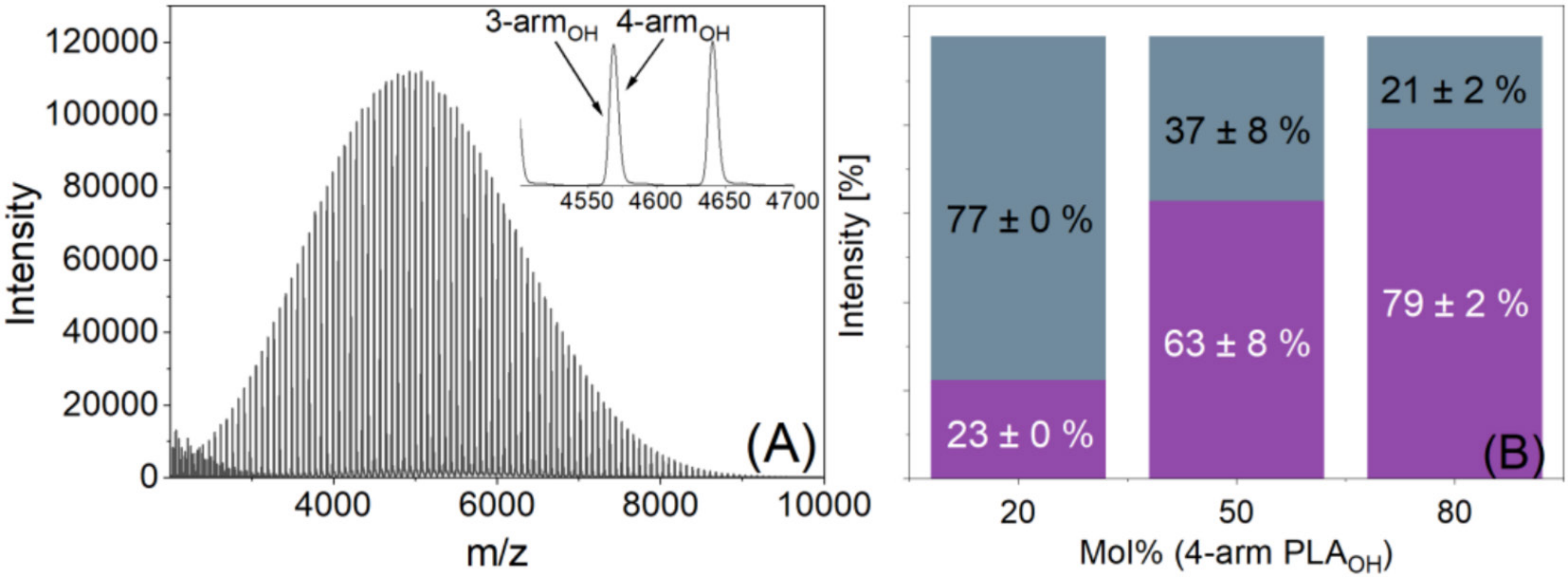
MALDI TOF mass spectrum (A) of an equimolar mixture of four‐arm and three‐arm PLA_OH_ and the resulting intensity ratios (B) of three binary blends of four‐arm (purple) and three‐arm (grey) PLA_OH_.

Unfortunately, it was difficult to distinguish between four‐arm and three‐arm PLA_OH_ in the applied linear mode of MALDI‐TOF MS due to the low mass difference of 2 Da between the signal series, as depicted in Figure 4A, which shows a MALDI spectrum of an equimolar blend of the PLA stars. Thus, the spectra had to be calibrated accurately. For binary blends of both stars, signals from linear PLA_OH_ that likely formed as a minor product during the synthesis of the PLA stars were used as the internal standard [23]. The internal standard was first used to correct the position of the mass peak of the stars and was then applied to binary mixtures of three‐arm and four‐arm PLA_OH_ with various compositions (20/80, 50/50, and 80/20). All blend compositions were measured in triplicate and are shown in different colors (see also caption of Figure S3). Figure 4B provides additional evidence that the intensity depends on the number of arms, as the four‐arm PLA_OH_ exhibits higher intensity than the three‐arm species. The high variance of the intensity values of the equimolar mixture mainly results from the limited number of data points in the spectra. Depending on the neighboring data points chosen, intensity differences of around 10% could result (also shown in Figure S3).

**FIGURE 5:**
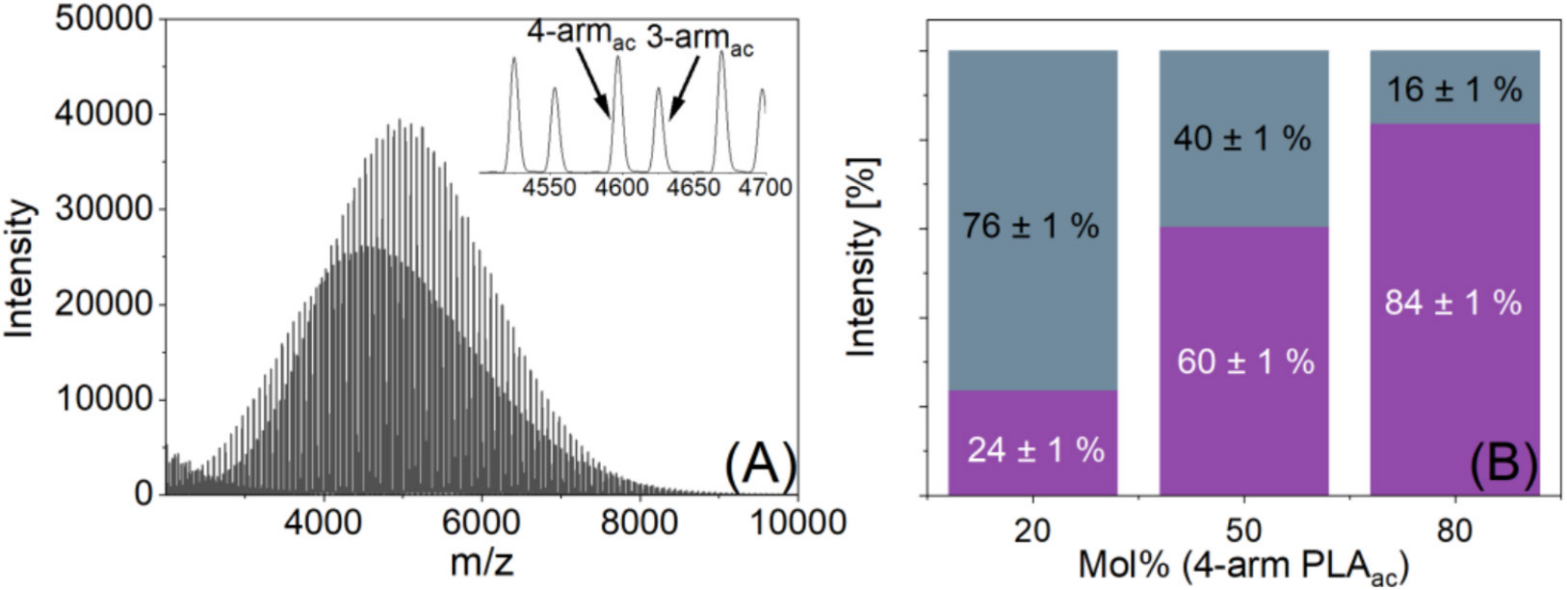
MALDI TOF mass spectrum (A) of an equimolar mixture of four‐arm and three‐arm PLA_ac_ and the resulting intensity ratios (B) of three binary blends of four‐arm (purple) and three‐arm (grey) PLA_ac_.

In contrast, binary blends of PLA star with acetylated end groups (three‐arm and four‐arm PLA_ac_) were successfully analyzed. The results showed that the four‐arm PLA_ac_ stars exhibited higher intensities than the three‐arm PLA_ac_ stars (Figure 5). Although the intensity differences were smaller than those between linear and starlike PLAs, this finding corroborates our previous results, demonstrating that intensity depends on the number of arms. Clearly, more arms provide more coordination sites or a higher density of coordination sites.

### 4.4 Ternary Blends of Linear and two Starlike PLA

Consequently, the next part of this study focused on investigating equimolar ternary blends consisting of both star polymers and the linear PLA. Experiments were performed with both acetyl and hydroxyl end groups. As mentioned before, signals of the two hydroxyl‐terminated PLA stars differed by only 2 Da, thus strongly overlapped. However, in the ternary blends, this problem could be easily overcome by accurately calibrating the mass spectra using one of the three polymers as an internal standard. The principal workflow is presented in Figure S4 in the Supporting Information. For this purpose, equimolar binary blends of lin‐PLA_OH_ with three‐arm, as well as with 4‐PLA star were initially prepared and analyzed with MALDI‐TOF MS. The linear PLA_OH_ was used as an internal standard (Figure S4A). After calibration, the *m/z* values of the stars in the binary mixtures could be used to create a calibration curve reflecting the composition of the unresolved star peak in the ternary mixtures (Figure S4B). The lower peak resolution in the linear mode (as shown in the insert of Figure S4C) resulted in a data point distance of 0.2 Da. This caused larger deviations of the peak maximum of two of the five curves (see Points 1 and 2 in Figure S4B).

**FIGURE 6:**
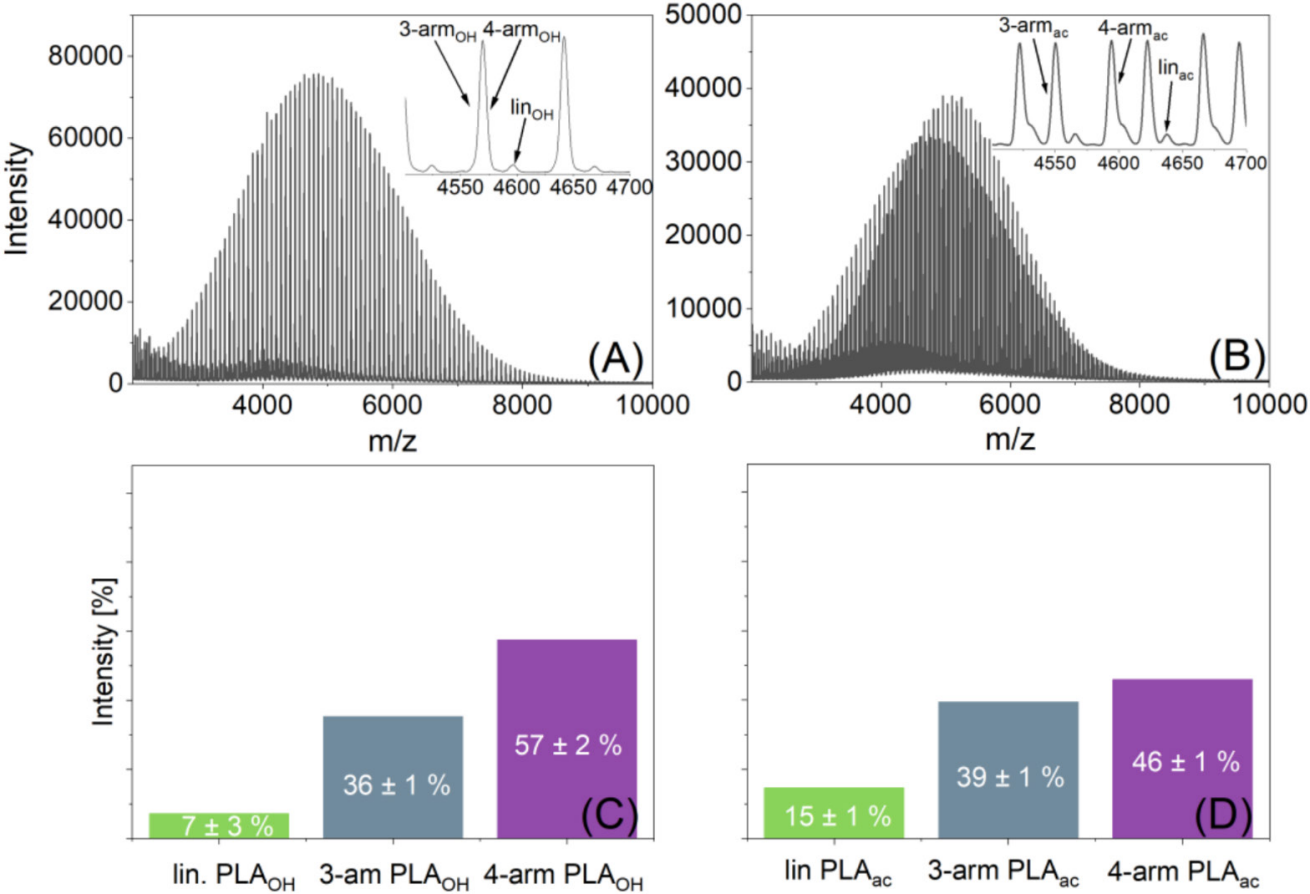
MALDI‐TOF mass spectra (A and B) and the resulting average intensity ratios of three repetitions (C and D) of equimolar ternary blends of lin‐PLA (green), three‐arm (grey), and four‐arm PLA (purple) with hydroxyl and acetyl end groups.

The MALDI spectra of the ternary blends are shown in Figure 6A,B. Figure 6C,D depict the resulting intensity ratios of the ternary hydroxyl‐ and acetyl‐terminated PLA blends calculated after such a calibration procedure. Once again, in both the hydroxyl‐ and acetyl‐terminated ternary blends, the measured intensity ratios deviate significantly from the theoretical equimolar value of 1:1:1. Consistent with previously observed behavior, the intensity of four‐arm PLA stars appears higher than that of three‐arm polymers and much higher than that of linear PLA. This effect seemed to be less pronounced for the acetylated species. While an additional effect of end‐group variation cannot be completely excluded, other chemical parameters, such as vaporability in the vacuum of the MALDI source, may also contribute.

### 4.5 Binary Blends of Linear and Starlike PEO

To gain a deeper insight, another polymer class (polyethylene oxide) was selected for the next part of the study. Hydroxyl end groups of PEO polymers can be easily modified in a similar way as described previously. Unfortunately, attempts to acquire well‐defined, commercially available, multi‐arm systems that cover a broad mass range mostly failed. To check the purity of such polymer stars, MALDI‐TOF MS and SEC analyses were performed (not shown), revealing the existence of up to four side products, mainly consisting of stars with a lower number of arms and linear polymers with a lower molecular weight. Thus, only a four‐arm poly (ethylene oxide) (PEO) with hydroxyl (OH) end groups was found to be suitable for our experiments. Another drawback was that the mass distributions of the two polymer species did not completely overlap. Therefore, an additional mass discrimination effect on the signal intensities of the higher mass four‐arm PEO must be considered.

**FIGURE 7:**
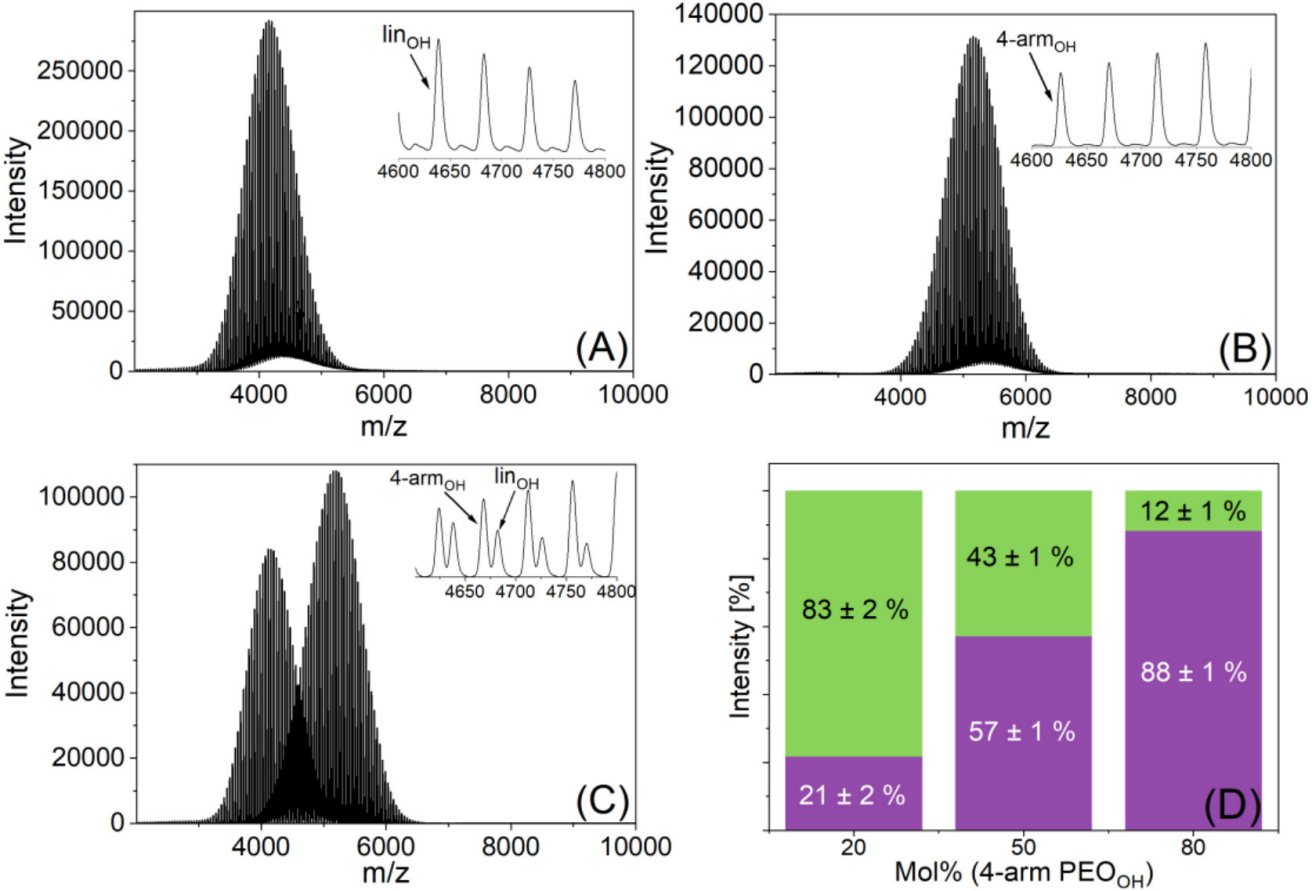
MALDI TOF mass spectra of linear (A), and four‐arm PEO_OH_ (B) and insets showing the region from *m/z* 4600 to 4800, mass spectrum of their equimolar blend (C), and the resulting intensity ratios for different mixing ratios (D), green = linear PEO, purple = four‐arm PEO.

**FIGURE 8:**
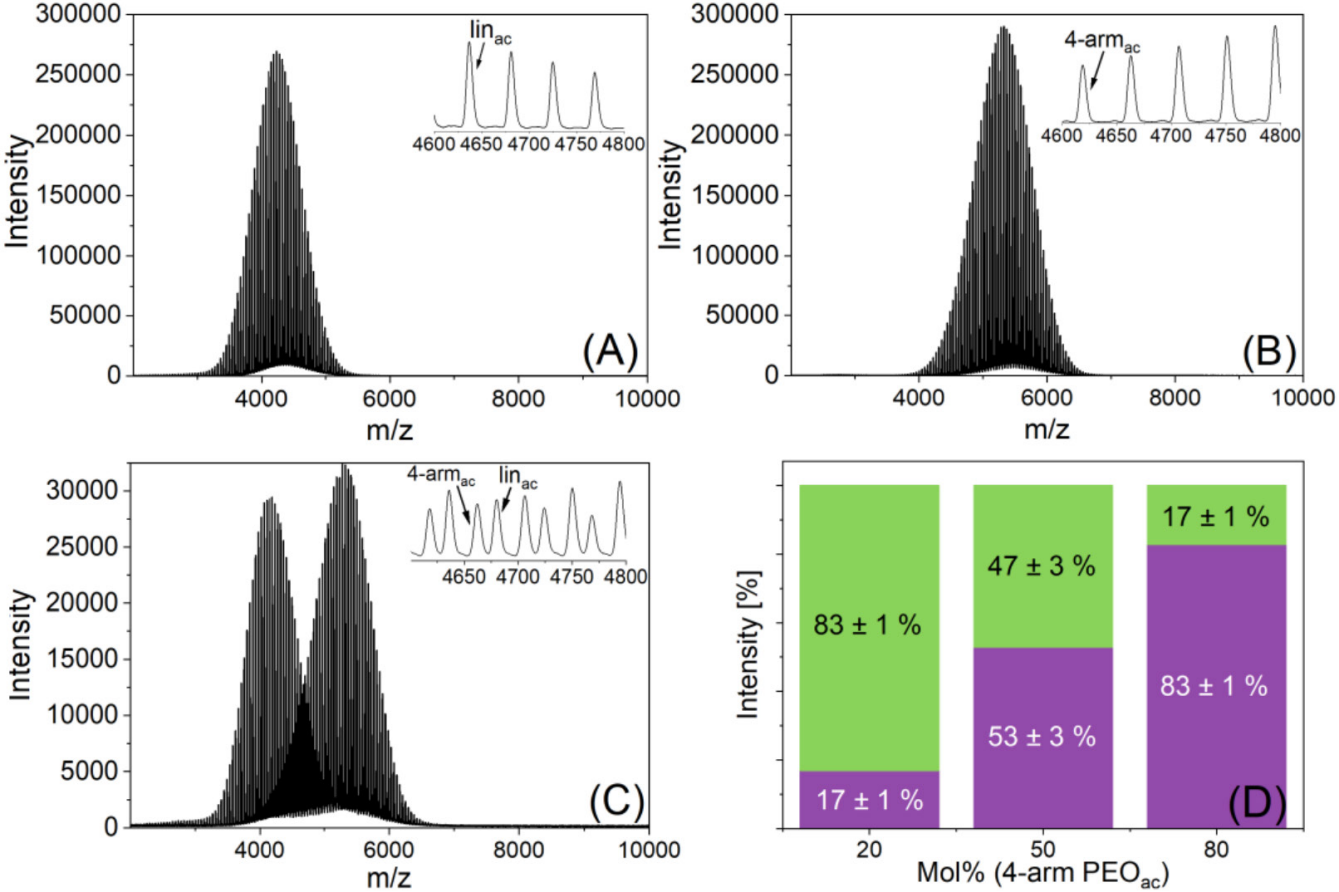
MALDI TOF mass spectra of linear (A), and four‐arm PEO_ac_ (B) and insets showing the region from *m/z* 4600 to 4800, mass spectrum of their equimolar blend (C), and the resulting intensity ratios for different mixing ratios (D), green = linear PEO, purple = four‐arm PEO.

Figure 7A,B show that the MALDI‐TOF mass spectra of the two polymers contain only one peak series. This was not surprising, as additional potassium salt adducts, seen with the linear PLA_OH_, could not be formed. The MALDI TOF mass spectra of the equimolar binary blend of the two PEOs is shown in Figure 7C. The results are summarized in Figure 7D together with those of the other blend compositions. These results showed a higher intensity of nearly 20% of the four‐arm PEO_OH_ compared with that of the linear PEO_OH_. To verify this finding, an acetylation of the PEO end groups was performed. The mass spectra of the functionalized lin‐ and four‐arm PEO_ac_ are shown in Figure 8A,B. The peak shift of 2 Da for lin‐PEO_ac_ clearly indicates acetylation on the hydroxyl‐terminated side. Four‐arm PEO_ac_ was completely functionalized as shown by the 8 Da peak shift in Figure 8B.

Again, a higher intensity of the four‐arm PEO compared with the linear PEO was found in three different binary blends for both hydroxyl‐ as well as acetyl‐terminated polymers, as seen in Figure 7D and Figure 8D.

As mentioned shortly before, blended linear and four‐arm PEOs have different molecular weights. During the MALDI process, higher masses are typically discriminated compared with lower mass species. This is caused by instrumental parameters like detector saturation by lower mass ions or ion‐focusing issues in the mass analyzer, as well as physicochemical factors related to the analyte like lower velocities due to higher molecular weights [4, 5, 9, 18, 60]. Shimada et al. attempted to quantitatively determine the mass discrimination effect of polystyrene (PS) polymers ionized with silver trifluoroacetate [61]. For this purpose, they separated uniform PS oligomers from commercial standard PS samples using preparative supercritical fluid chromatography (SFC). The researchers demonstrated that it was possible to calibrate mass‐dependent intensities in MALDI‐TOF mass spectra up to *m/z* 2500. Based on their calculations, an intensity difference of approximately 20% can be assumed for two polymer homologues with a molecular weight difference of 1000 Da at moderate laser power. In other words, if the molecular masses were similar, the relative intensity of the stars in the blend with linear PEOs would be even higher. However, it was not possible to make a clear assumption at this point. Nevertheless, these results further confirm the influence of the polymer architecture on mass spectral signal intensities.

### 4.6 Calculation of Binding Energies of PEO/K^+^

Binding energy calculations were performed using ORCA to determine the strength of the interaction between a PEG molecule and a potassium (K^+^) ion. In these calculations, the binding energy (*E*_bind_) is obtained using the following equation:

$${\text{𝐸}}_{\text{bind}}={E_{\left[\text{polymer}-K\right]}}^{+}-E_{\text{polymer}}-{E_{K}}^{+}$$

Here, *E*_[polymer‐K]_^+^ is the total DFT energy of the polymer‐K^+^ complex, and the other terms are the energies of the isolated components. A more negative binding energy indicates a stronger interaction and greater stability of the cation‐polymer complex.

**FIGURE 9:**
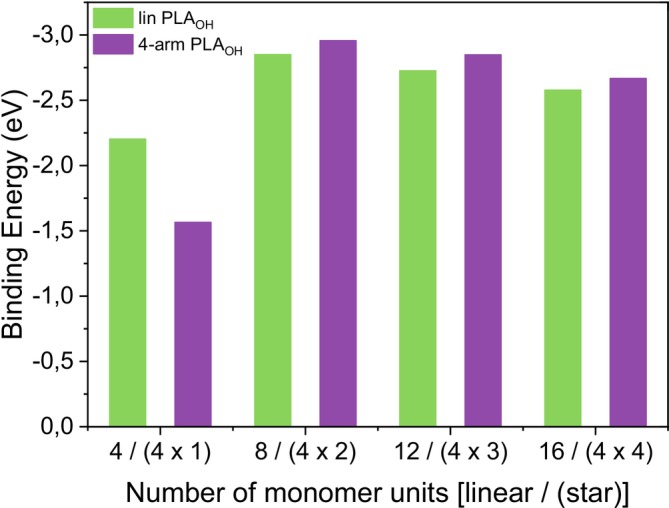
Calculated binding energies of the [polymer/K]^+^ adduct ions of linear and four‐arm PEO depending on the number of monomer units.

Because the time required to calculate a larger number of monomer units increased exponentially, calculations were only performed up to 16 units. In Figure 9, the binding energy (in electron volt) was plotted versus the number of monomer units (*n*) for the two polymer types (linear and four‐arm). To ensure a fair comparison of binding energies, the same number of monomers was used for both polymers. Therefore, *n* = 4 represents either a linear structure with four monomer units or a four‐arm star with one monomer unit attached to each arm. Similarly, *n* = 16 describes a linear polymer chain consisting of 16 units and an ideal four‐arm star, with each arm consisting of four monomer units. Of course, other combinations of the arm length distribution could not be excluded. However, as shown in a previous investigation, the chromatographic separation by gradient elution liquid adsorption chromatography (GELAC) resulted in a clear separation of linear chains from those of the stars [23]. This makes extreme differences in the arm length of stars seem unlikely. As can be seen from Figure 9, the binding energy depends on both the polymer topology and the number of linkers. For *n* = 4 (i.e., fewer linkers), linear polymers tend to bind more strongly to K^+^ likely due to compact coordination sites. As the number of linkers increases, star‐shaped polymers exhibit stronger binding affinities (more negative *E*_bind_), suggesting that branching enhances the spatial accessibility and charge distribution around the metal ion compared with linear polymers. Though the difference in binding energies between star polymers and linear polymers is only a few percent, it is notable that this difference remains relatively constant for longer polymer chains. Thus, the results from ORCA may help correlate theoretical binding strengths with MALDI ionization efficiencies and explain why specific polymer structures form more stable adducts and produce stronger signals in mass spectra.

## 5 Conclusions

Our investigation revealed that polylactide and polyethylene oxide stars exhibited significantly higher intensities than their linear counterparts in equimolar blends. The same effect was observed in blends of these polymers with acetylated end groups. This may be due to an increased abundance or density of oxygen‐containing binding sites available for adduct formation during ionization. This process is similar to the coordination and complexation of ions observed in solution. Since MALDI ionization is a competitive process, the increased availability of these binding sites in polymer stars appears advantageous.

Although similar results were obtained after acetylation of the hydroxyl end groups, an influence of the end groups on the ionization cannot be completely ruled out. However, this assumes that hydroxyl and carboxylic functional groups have a similar ionization tendency. This does not change the statement that star‐shaped polymers have significantly higher peak intensities than linear polymers. It only indicates that the site where ionization occurs is still unknown.

Calculations of binding energies revealed that the interaction between a star‐shaped polyethylene glycol (PEG) and a potassium (K^+^) ion is slightly stronger than that between linear PEGs and K^+^ ions.

Further, MALDI‐MS experiments will involve star‐shaped polymers with a higher number of arms than those used in our study, as well as dendrimers. Likewise, comparing star‐shaped polymers with polymers of other structures (e.g., comb‐shaped or cyclic) could shed more light on how polymer architecture influences mass spectrum intensity.

## Supporting information


**Table S1:** Molar masses of the analyzed polylactide and poly (ethylene oxide) polymers.
**Figure S1:** Mass spectra of linear PLA_ac_ (A), three‐arm PLA_ac_ (B), and four‐arm PLA_ac_ (C); The number sign indicates ca. 3% of incompletely functionalized four‐arm PLA_ac_.
**Figure S2:** MALDI TOF mass spectra of equimolar binary blends of linear PLA_ac_ with three‐arm PLA_ac_ (A) and four‐arm PLA_ac_ (B); the number sign indicates ca. 3% of incompletely functionalized 4‐arm PLA_ac_; the asterisk indicates the additional COOK series of the linear PLA_ac_.
**Figure S3:** Calibration of three‐arm (black) and four‐arm (blue) PLA_OH_ via single samples with internal standard linear PLA_OH_ (A), signals of the single samples (triplicates) applied for calibration (B), all investigated mixtures of four‐arm/three‐arm PLA_OH_ with the mixing ratios 20/80 (black curve for samples 1, 2, 3), 50/50 (blue curve for samples 4, 5, 6), and 80/20 (grey curve for samples 7, 8, 9) (C), and the resulting calibration curve (D).
**Figure S4:** Calibration of three‐arm and four‐arm PLA_OH_ via equimolar blends with internal standard linear PLA_OH_ (A), the resulting calibration curve (B), the mass spectra of all investigated samples of equimolar blend of both PLA stars with hydroxide end groups (C), and the resulting average intensity ratios of the applied method (D).

## Data Availability

The data that support the findings of this study are openly available in Zenodo at https://zenodo.org/uploads/17598552.
