# Supplementary material for: Quantitative Analysis of Polymers by MALDI‐TOF Mass Spectrometry: Correlation Between Signal Intensity and Arm Number
Source: J Mass Spectrom. 2026 Jan 1;61(1):e70023. doi: 10.1002/jms.70023 (PMC12757704; doi:10.1002/jms.70023)
Supplement: Supplementary file 1 — Table S1: Molar masses of the analyzed polylactide and poly (ethylene oxide) polymers. Figure S1: Mass spectra of linear PLAac (A), three‐arm PLAac (B), and four‐arm PLAac (C); The number sign indicates ca. 3% of incompletely functionalized four‐arm PLAac. Figure S2: MALDI TOF mass spectra of equimolar binary blends of linear PLAac with three‐arm PLAac (A) and four‐arm PLAac (B); the number sign indicates ca. 3% of incompletely functionalized 4‐arm PLAac; the asterisk indicates the additional COOK series of the linear PLAac. Figure S3: Calibration of three‐arm (black) and four‐arm (blue) PLAOH via single samples with internal standard linear PLAOH (A), signals of the single samples (triplicates) applied for calibration (B), all investigated mixtures of four‐arm/three‐arm PLAOH with the mixing ratios 20/80 (black curve for samples 1, 2, 3), 50/50 (blue curve for samples 4, 5, 6), and 80/20 (grey curve for samples 7, 8, 9) (C), and the resulting calibration curve (D). Figure S4: Calibration of three‐arm and four‐arm PLAOH via equimolar blends with internal standard linear PLAOH (A), the resulting calibration curve (B), the mass spectra of all investigated samples of equimolar blend of both PLA stars with hydroxide end groups (C), and the resulting average intensity ratios of the applied method (D). [file JMS-61-e70023-s001.docx]

**Supporting Information**

**to**

**Quantitative Analysis of Polymers by MALDI-TOF Mass Spectrometry: Correlation between Signal Intensity and Arm Number**

Mete-Sungur Dalgic, Sourabh Kumar and Steffen M. Weidner*

Bundesanstalt für Materialforschung und -prüfung -BAM, Richard-Willstätter-Str. 11, D-12489 Berlin, Germany

* Author for correspondence: steffen.weidner@bam.de

Table 1 Molar masses of the analyzed polylactide and poly(ethylene oxide) polymers.

| Sample | M_p_ [kg mol^-1^] |
| --- | --- |
| Lin. PLA_OH_ | 4.1 |
| Lin. PLA_ac_ | 4.1 |
| 3-arm PLA_OH_ | 4.7 |
| 3-arm PLA_ac_ | 5.3 |
| 4-arm PLA_OH_ | 4.8 |
| 4-arm PLA_ac_ | 5.4 |
| Lin. PEO_OH_ | 4.1 |
| Lin. PEO_ac_ | 4.2 |
| 4-arm PEO_OH_ | 5.2 |
| 4-arm PEO_ac_ | 5.3 |

**Figure S1** Mass spectra of linear PLA_ac_ ((A)), 3-arm PLA_ac_ ((B)), and 4-arm PLA_ac_ ((C)), (#) indicates ca. 3 % of incompletely functionalized 4-arm PLA_ac_.

**Figure S2** MALDI TOF mass spectra of equimolar binary blends of linear PLA_ac_ with 3-arm PLA_ac_ (A) and 4-arm PLA_ac_ (B), (#) indicates ca. 3 % of incompletely functionalized 4-arm PLA_ac_, (*) indicates the additional COOK series of the linear PLA_ac_


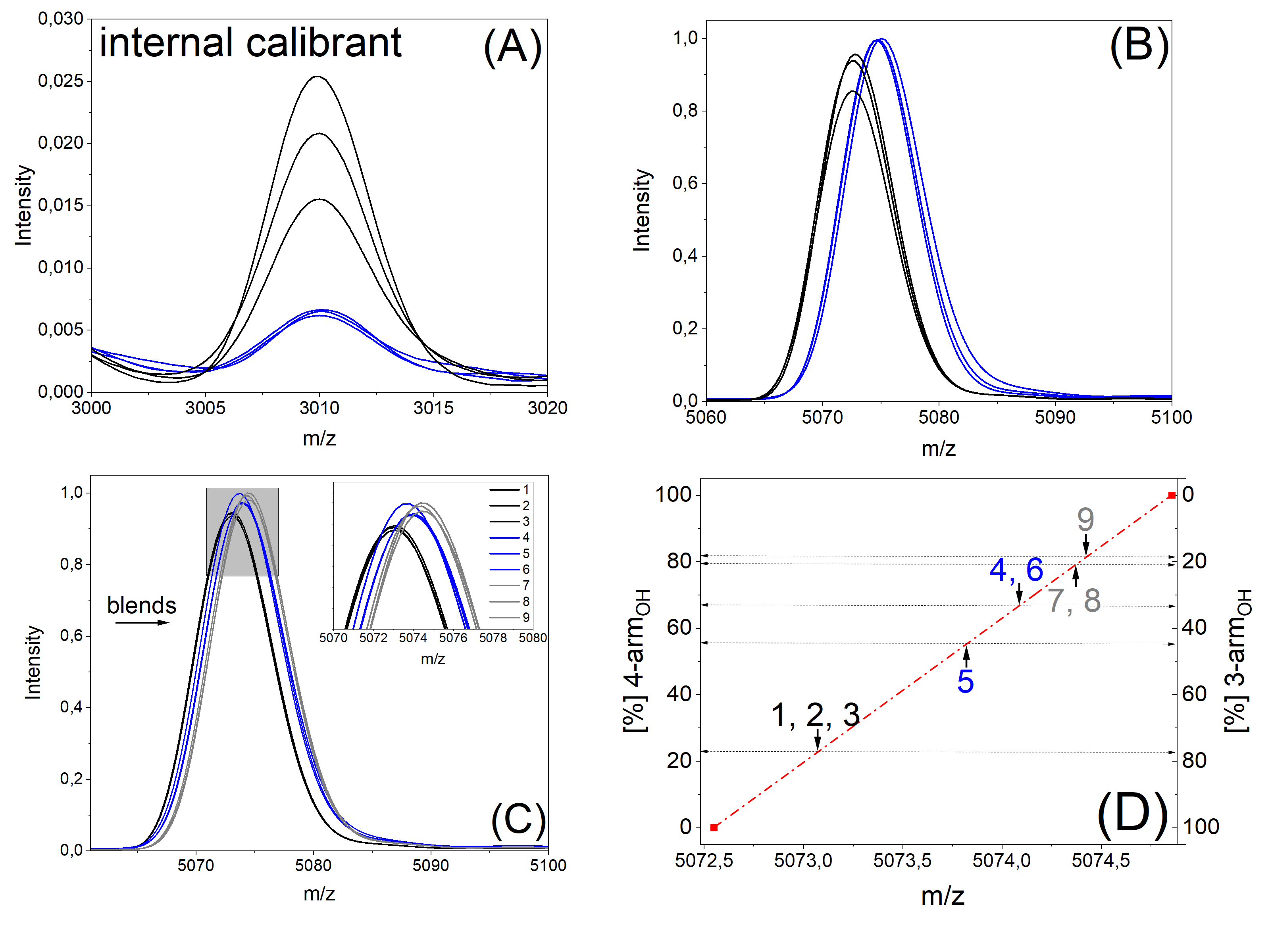
**Figure S3** Calibration of 3-arm (black) and 4-arm (blue) PLA_OH_ via single samples with internal standard linear PLA_OH_ (A), signals of the single samples (triplicates) applied for calibration (B), all investigated mixtures of 4-arm/3-arm PLA_OH_ with the mixing ratios 20/80 (black curve for samples 1,2,3), 50/50 (blue curve for samples 4, 5, 6), and 80/20 (grey curve for samples 7, 8, 9) (C), and the resulting calibration curve (D).


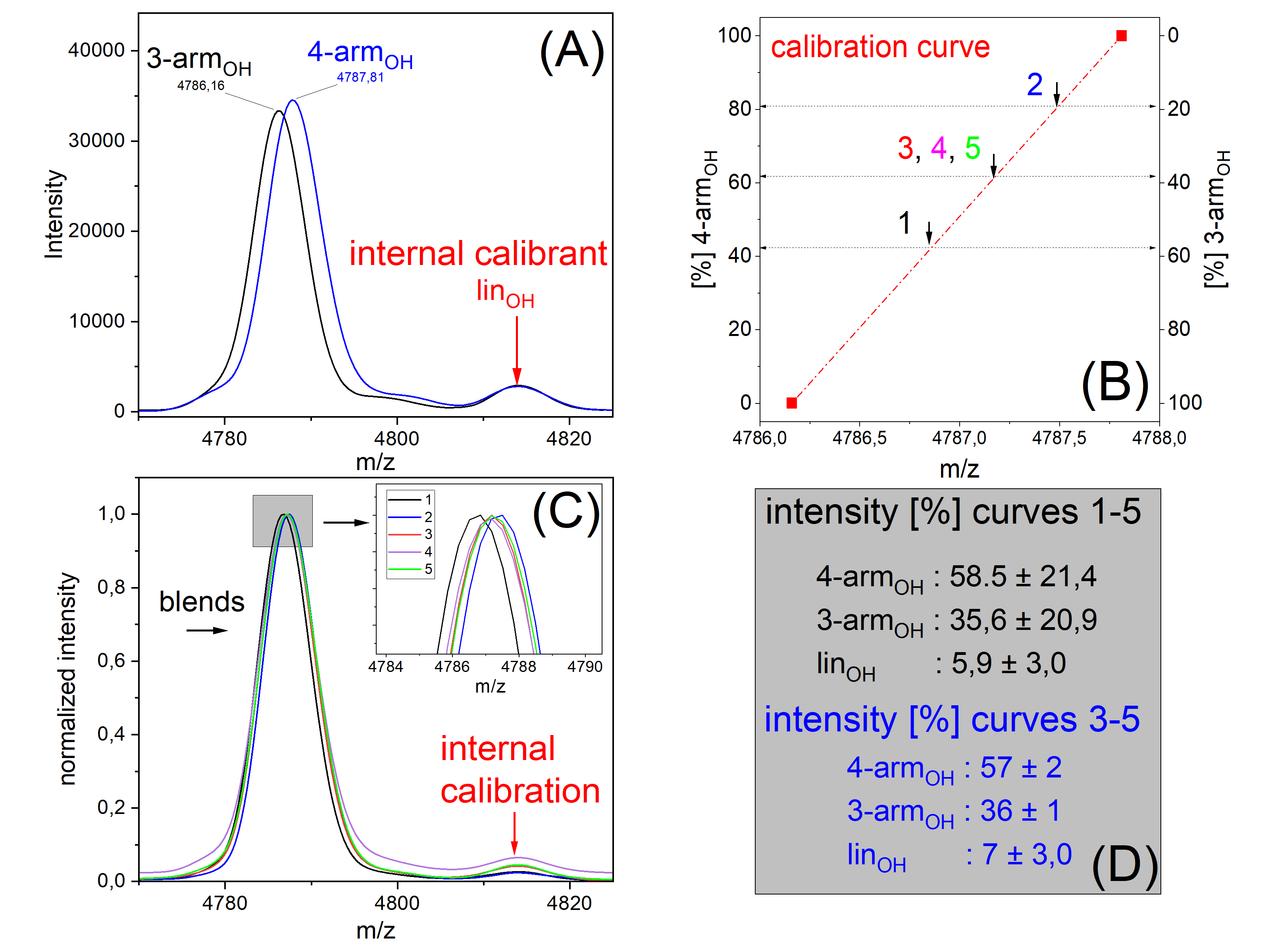


**Figure S4** Calibration of 3-arm and 4-arm PLA_OH_ via equimolar blends with internal standard linear PLA_OH_ ((A)), the resulting calibration curve (B), the mass spectra of all investigated samples of equimolar blend of both PLA stars with hydroxide end groups ((C)), and the resulting average intensity ratios of the applied method ((D)).
